# Modulating Substrate Specificity of *Rhizobium* sp. Histamine Dehydrogenase through Protein Engineering for Food Quality Applications

**DOI:** 10.3390/molecules28093748

**Published:** 2023-04-26

**Authors:** Karen Rodríguez-Núñez, Alejandra Cortés-Monroy, Marcela Serey, Yunus Ensari, Mehdi D. Davari, Claudia Bernal, Ronny Martinez

**Affiliations:** 1 Departamento de Ingeniería en Alimentos, Universidad de La Serena, Av. Raúl Bitrán 1305, La Serena 1720010, Chile; 2 Department of Bioengineering, Faculty of Engineering and Architecture, Kafkas University, Kars 36000, Turkey; 3 Department of Bioorganic Chemistry, Leibniz Institute of Plant Biochemistry, Weinberg 3, D-06120 Halle, Germany; 4 Instituto de Investigación Multidisciplinaria en Ciencia y Tecnología, Universidad de La Serena, Av. Raúl Bitrán 1305, La Serena 1720010, Chile

**Keywords:** enzyme engineering, histamine, dehydrogenase, agmatine, diaminopropane, protein design

## Abstract

Histamine is a biogenic amine found in fish-derived and fermented food products with physiological relevance since its concentration is proportional to food spoilage and health risk for sensitive consumers. There are various analytical methods for histamine quantification from food samples; however, a simple and quick enzymatic detection and quantification method is highly desirable. Histamine dehydrogenase (HDH) is a candidate for enzymatic histamine detection; however, other biogenic amines can change its activity or produce false positive results with an observed substrate inhibition at higher concentrations. In this work, we studied the effect of site saturation mutagenesis in *Rhizobium* sp. Histamine Dehydrogenase (Rsp HDH) in nine amino acid positions selected through structural alignment analysis, substrate docking, and proximity to the proposed histamine-binding site. The resulting libraries were screened for histamine and agmatine activity. Variants from two libraries (positions 72 and 110) showed improved histamine/agmatine activity ratio, decreased substrate inhibition, and maintained thermal resistance. In addition, activity characterization of the identified Phe72Thr and Asn110Val HDH variants showed a clear substrate inhibition curve for histamine and modified kinetic parameters. The observed maximum velocity (*V_max_*) increased for variant Phe72Thr at the cost of an increased value for the Michaelis–Menten constant (*K_m_*) for histamine. The increased *K_m_* value, decreased substrate inhibition, and biogenic amine interference observed for variant Phe72Thr support a tradeoff between substrate affinity and substrate inhibition in the catalytic mechanism of HDHs. Considering this tradeoff for future enzyme engineering of HDH could lead to breakthroughs in performance increases and understanding of this enzyme class.

## 1. Introduction

Histamine is a biogenic amine synthesized through enzymatic decarboxylation of histidine and plays various physiological and metabolic roles [1]. It is produced and stored in basophils and mast cells, and is also found in gastric cells that trigger gastric acid release and histaminergic neurons. In addition, during inflammation initiated by an allergen, histamine is released by mast cells, inducing allergic or inflammatory reactions and modulating innate and adaptative immune responses [2].

Histamine is also found in low concentrations in food and beverages, particularly in some fruits and vegetables, seafood, fish, matured cheese, and other fermented food [3]. In rotten food, however, toxic levels of histamine (>500 mg/kg) can lead to food poisoning, often a direct result of bacterial growth in the food matrix.

Histamine toxicity, also called “scombroid fish poisoning”, is linked to microbial protease and decarboxylase activity in spoiled or rotten fish and other high protein/high histidine food matrices.

Furthermore, histamine buildup indicates product freshness and proper storage, especially in fish products. Therefore, histamine detection and quantification are crucial for food safety and quality assurance [3]. Additionally, early histamine detection is a critical process for niche applications such as low histamine food products for the histamine-hypersensitive population, which—as for other food intolerances—is steadily increasing, currently affecting approximately 1% of the population [4].

Current histamine analysis methods are based on chromatographic analytics for biogenic amines, such as thin-layer chromatography and high-performance liquid chromatography (TLC, HPLC), which have been well-summarized [5]. Histamine can be separated through HPLC using a C18 reversed-phase column and an acetonitrile−water gradient, which is also applied for the detection of biogenic amines, usually employing pre- or postcolumn derivatization using o-phtalaldehyde (OPA) or dansyl chloride [6] under alkaline and reducing conditions for colorimetric determination or using o-phtaldehyde (OPT) for fluorogenic detection. HPLC separation coupled with high-resolution mass spectrometry allows for decreasing the detection limit for histamine, reaching 5 mg/kg in fish meat [5] and 0.07 ng/mL in baby food [6]. Despite achieving impressive detection methods, analytical methods for quantifying histamine (and other biogenic amines) require sample concentration, purification, advanced instrumentation, and specialized operators.

Alternatively, enzyme-based detection of histamine is a promising alternative to analytic methods. Two main types of enzymatic approaches have been described: electro-enzymatic and colorimetric. Histamine oxidases (HOD, EC 1.4.3.6) catalyze the oxidative deamination of primary amines (e.g., histamine), releasing ammonia and hydrogen peroxide. For example, the development of enzyme-coated electrodes allowed the electrochemical detection of various biogenic amines, including histamine, allowing the detection of 3 µM histamine [7].

**Figure 1:**
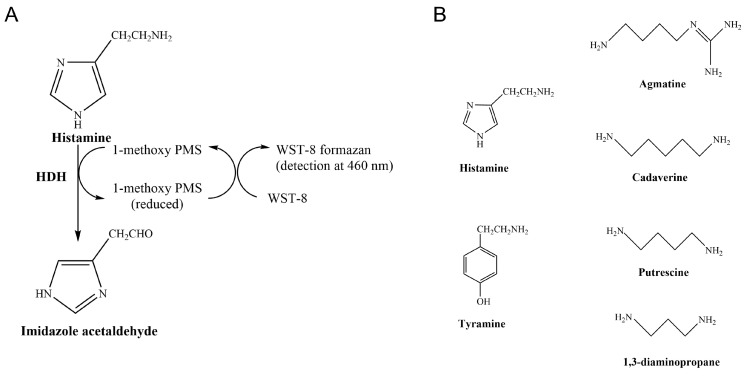
(**A**) Chromogenic assay for histamine dehydrogenase (HDH) [8]; histamine is decomposed in the presence of 1-methoxy PMS to produce reduced 1-methoxy PMS and transfer electrons to a chromogenic tetrazolium salt (WST-8, or MTT), absorbing at 460 nm. (**B**) Structure of the relevant biogenic amines for this work.

In enzyme-based chromogenic assays, histamine is degraded by Histamine Dehydrogenase (HDH, EC 1.4.9.1) in the presence of an electron carrier (1-methoxy-5-methylphenazinium methylsulfate, 1-PMS), producing reduced 1-methoxy PMS, transferring electrons to a chromogenic tetrazolium salt (WST-8, or MTT), and the product can be measured at 460 nm (Figure 1A). These colorimetric assays present a valuable opportunity for developing affordable histamine detection kits that could be routinely used for food quality assurance and control.

HDHs have activity against cadaverine, putrescine, tyramine, tryptamine, spermine, and spermidine (Figure 1B). *Rhizobium* sp. HDH (Rsp HDH) showed agmatine oxidation at a rate of 10% that of histamine [8] and 1,3-Diaminopropane at a 13% rate of histamine [9]. In contrast, HDH from *Nocardioides simplex* (HDH-N) converted putrescine at 30% the rate exhibited with histamine [10]. Available HDHs also suffer from substrate inhibition, especially from the primary substrate, histamine. The latter affects enzyme performance in complex samples, hindering the accuracy and specificity of enzyme-based histamine detection and quantification methods [11,12]. Thus, HDHs with high histamine specificity and reduced substrate inhibition are needed to develop specific histamine colorimetric methods for the food industry.

Enzyme limitations such as substrate specificity profiles and inhibition can be improved using protein engineering. Protein engineering strategies aim to obtain enzymes with higher performance than the starting variant [13]. Directed evolution is the most used strategy in enzyme engineering [14,15]. Improved variants can be generated and identified using iterative cycles of gene diversity generation and high throughput screening. In addition, specific enzyme residues and residues based on in silico, structural, and computational chemistry approaches can be targeted through rational and semi-rational protein engineering approaches [13,14,15,16].

In this work, we studied the effect of site saturation mutagenesis in selected amino acid residues near the proposed histamine-binding site in *Rhizobium* sp. Histamine Dehydrogenase (Rsp HDH) to identify novel structure–function relationships contributing to decreasing its activity against other biogenic amines and reducing substrate inhibition. For this, we performed in silico structural analysis of Rsp HDH, selected amino acid positions for site saturation mutagenesis (SSM), screened for histamine and agmatine activity using a microtiter plate format colorimetric assay, selected for variants with improved histamine/agmatine activity ratios, and maintained thermal resistance. Then, selected variants were characterized, and their structure–function changes were studied in silico.

## 2. Results and Discussion

The results are presented as the structural analysis of Rsp HDH to select amino acid sites for SSM, construction of the SSM libraries, library screening, amino acid substitution combination, variant characterization regarding substrate activity and thermal resistance compared against WT Rsp HDH, and structure–function analysis of the selected variants.

**Figure 2:**
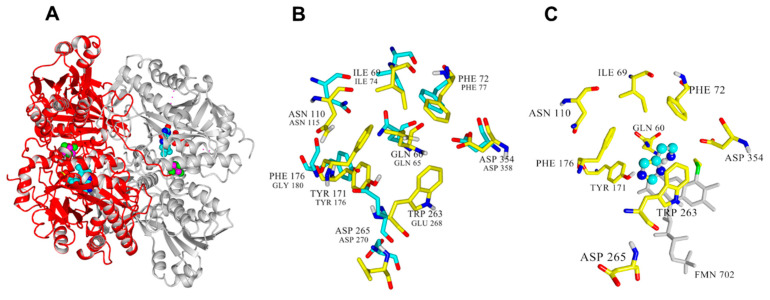
(**A**) Structure of the homodimer of *Rhizobium* sp. Histamine Dehydrogenase (PDB ID: 6DE6). (**B**) Selected positions for site saturation mutagenesis in *Rsp* HDH (yellow, bigger font) and their corresponding amino acid residue in *N. simplex* (cyan, smaller font). (**C**) The lowest energy pose obtained for histamine docking in *Rsp* HDH. The labeled amino acid residues are potentially involved in the interaction or stabilization of the substrate.

The crystal structures for *Nocardioides simplex* HDH (PDB ID: 3K30) [17] and *Rhizobium* sp. HDH (PDB ID: 6DE6) are available (Figure 2A). They are structurally similar [10,17], with each chain containing a 6-S-Cys-FMN and [4Fe-4S] as redox-active cofactors and a non-covalently bound ADP molecule [18]. In addition, *Nocardioides simplex* HDH has proposed histamine binding sites (Gln65, Ile74, Phe77, Glu79, Asn115, Tyr181, Trp267, Asp358), which are potential targets for mutagenesis studies [10,17]. 

The structure of Rsp HDH (693 aa, Uniprot Q60I59) was used as a starting point for this study since it has a more histamine-specific substrate spectrum and high thermal resistance [8,9], making it an attractive starting point for protein engineering. However, its structure was released in 2019, with a yet-to-be-released accompanying research publication providing more detailed information. For analysis, the structure was energy minimized using YASARA Structure [19,20], and the resulting structure was aligned with the structure of the Histamine Dehydrogenase of *Nocardiopsis simplex* (PDB ID: 3K30) (Figure 2B), where residues involved in catalysis and substrate binding have been described [15]. This structural alignment allowed us to identify the corresponding residues in Rsp HDH potentially involved in catalysis and substrate [17,21]. Thus, residues Gln60, Glu74, Phe72, Tyr171, His174, Arg225, Trp263, and Asp265 in Rsp HDH formed the active site. Furthermore, the catalytic triad in Rsp HDH comprises Tyr171, His174, and Asp265. To identify amino acid positions probably having direct contact with the substrate, mechanism-based [22,23] molecular docking experiments were performed using the minimized structure of Rsp HDH.

Docking results show the best catalytically competent docking pose, with a calculated binding energy of −4.37 kcal/mol (Figure S1). The resulting distance between the N atom of histamine and the N5 of FMN was 2.655 Å (Table S1). Furthermore, the imidazole ring of histamine was stabilized via a pi-stacking interaction with Trp263, His174, and Tyr171 (Figure 2C). The imidazole ring of histamine interacted with F176 and Tyr171, whereas the ethylamine group could form hydrophobic interactions with the residues Phe72, Glu74, Cys30, and Trp263. These observations suggest that hydrophobic interactions modulate substrate binding in Rsp HDH.

Additionally, the residues aligning in active site cavities and tunnels were identified using Caver web tool 1.0 [24]. In total, 66 lining residues with Pocket relevance scores of 100%, volume 4255 A^3^, and draggability: 0.63 values were identified (Figure S2 and Table S2). Furthermore, residues Gln60, Ile69, Phe72, Glu74, Asn110, Phe131, Tyr171, Ala173, His174, Gly175, Phe176, Thr262, Trp263, Asp354, Met355, and Leu567 were identified by the Caver web tool as the residues constitute the tunnel one, which seems important for the substrate transport to the active site of the *Rsp* HDH (Figure S3 and Table S3). Finally, integrating the structural alignment, docking results, distance from histamine, and cavity analysis, residues Gln60, Ile69, Phe72, Asn110, Tyr171, Phe176, Trp263, Asp265, and Asp354 were selected as the target for site saturation mutagenesis to explore their effect in HDH activity.

The SSM libraries were generated using Quick change PCR [25], introducing NNK degenerate codons through specifically designed primers for each library (Table S4), and the libraries were transformed into *E. coli* BL21 Gold (DE3) cells for activity screening (~90 colonies per library), approximately 3× the necessary amount of colonies needed to cover the codon diversity [16]. The correct construction for each library was confirmed using DNA sequencing (through diversity introduction in the specific codon for the PCR product). Overall, ~1000 colonies were picked from transformation agar plates directly to stock microtiter plates (MTPs).

The screening assay was based on a colorimetric assay where the biogenic amine substrate was degraded by HDH, producing reduced 1-methoxy PMS and transferring electrons to a chromogenic tetrazolium salt (WST-8), resulting in an increase in absorbance at 460 nm [8], adapted to the 96-well MTP format (Figure 1A). The assay was validated by growing and inducing 96 *E. coli* clones producing WT Rsp HDH in an MTP and obtaining a standard deviation for the measured activity value below 15%, which is acceptable for mutant library screening [16].

For SSM library screening, two activity measurements were performed per clone, one using histamine and another using agmatine as substrate (Figure 1B), both at 320 µM. HDH from *N. simplex* has shown activity against histamine, agmatine, and putrescine, with *K_m_* values of 31, 37, and 1280 mM, respectively [18]. On the other hand, HDH for *Rhizobium* sp., despite having high substrate specificity [9], showed oxidation of agmatine and 1,3-diaminopropane at 10% and 13% the rate of histamine oxidation, respectively. Thus, increased substrate specificity is desired for histamine detection and quantification applications [9,18].

Screening for histamine activity allowed us to keep track of the main activity of the variants, whereas measuring agmatine activity allowed for calculating the histamine/agmatine activity ratio, describing changes in substrate specificity independently from the total activity measured for each clone.

**Figure 3:**
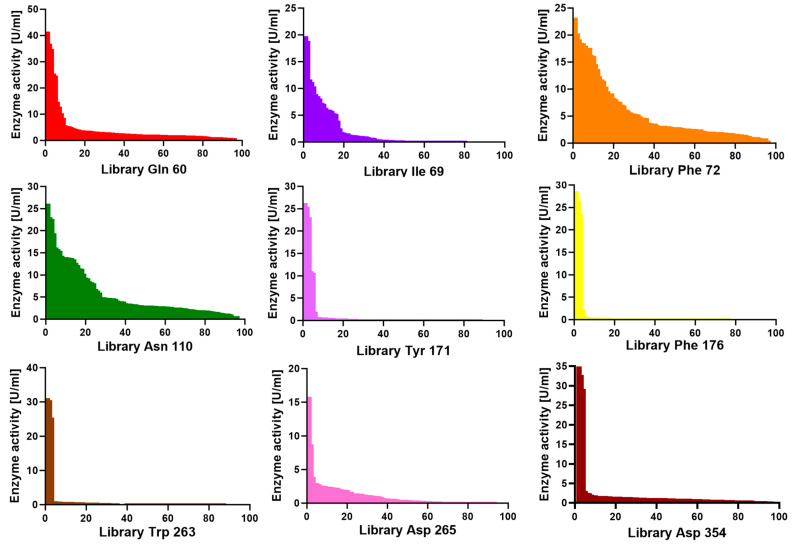
Histamine activity profile across the variant population for sample plates of the screened SSM libraries of Rsp HDH. The histamine activity of the clones has been plotted in decreasing order for each library. Positions Tyr171, Phe176, Trp263, Asp 265, and Asp 354 seem more sensitive to amino acid substitutions since histamine activity is lost for most screened clones. An intermediate situation was observed for positions Gln60 and Ile69, where active clones were identified but with much lower histamine dehydrogenase activity than WT Rsp HDH. On the other hand, for libraries Phe72 and Asn110, a wide range of activity values were found, suggesting these positions are less sensitive for amino acid substitutions in the case of histamine as substrate.

After screening all nine SSM libraries, we observed differences between the histamine activity profile across the variant population. Figure 3 shows screening plates where the measured histamine activity of the clones has been plotted in decreasing order. For libraries Tyr171, Phe176, Trp263, Asp265, and Asp354, the vast majority of the clones in the plate had no activity, and all identified active clones had no amino acid substitutions. Evolutionary conservation analysis of each residue was performed using Consurf [26] and UET servers [27] (Figures S4 and S5). Consurf analysis revealed that Tyr171, Phe176, and Trp263 scored nine, seven, and nine, respectively, showing that they are highly conserved residues (Table S5).

This amino acid substitution sensitivity suggests these positions are critical for enzyme function. The structural equivalents of Tyr171 and Asp265 in *N. simplex* HDH (Tyr176 and Asp270) are described as part of the catalytic triad [17]. On the other hand, positions Trp 263 and Phe 176 did not align structurally with relevant residues in *N. simplex* HDH. However, position Trp 267 of the latter was reported to be part of an “aromatic bowl” for substrate binding [17]; interestingly, in the work of Tsutsumi et al. [21], three positions in *N. simplex* HDH close to the structural equivalents of Phe176 and Trp263 in Rsp HDH, namely Tyr181, Gly269, and Asp270 (numbered Tyr 180, Gly268, and Asp269 in the cited work), were subjected to site-directed mutagenesis to Phe180, Asp269, and Cys269, reporting changes in the redox potential of the enzyme, leading to changes in the kinetic parameters and substrate inhibition profiles.

An intermediate situation was observed for positions Gln60 and Ile69, where active clones were identified but with much lower histamine dehydrogenase activity than WT Rsp HDH, suggesting these amino acid positions are somewhat optimized for histamine already. Gln60 and Ile69 were identified as less conserved, with Consurf scores of seven and six, respectively (Figures S4 and S5 and Table S5).

On the other hand, for libraries Phe72 and Asn110, a wide range of activity values were found, suggesting these positions are less sensitive for amino acid substitutions in the case of histamine as substrate and probably not yet optimized for the screened criteria. Additionally, these two residues were identified as variable residues by Consurf analysis (Figures S4 and S5 and Table S5). 

Since variants from mutagenesis libraries at positions 72 and 110 were selected for increased histamine/agmatine activity ratio, a double SSM library (Phe72/Asn110) was constructed for Rsp HDH. In addition, the increased theoretical diversity from introducing two simultaneous NNK codons in the gene (32 × 32 codon combinations) meant that more than 3000 variants needed to be screened to reach at least 95% diversity coverage.

We screened the double SSM library for increased histamine/agmatine activity ratio using 320 µM for histamine and 1000 µM agmatine. Additionally, we included another screening measurement using 1000 µM histamine to assess substrate inhibition, which is reported for Histamine Dehydrogenases, specifically for *N. simplex* and Rsp HDH [9,18,21]. Finally, since combining two amino acid substitutions could reduce enzyme stability or thermal resistance, we included an additional measurement by incubating the enzyme-containing lysate at 60 °C for 20 min, followed by a centrifugation step to ensure the selected variants would have a comparable thermal resistance to WT Rsp HDH, which might be necessary for further technical application of the enzyme.

**Figure 4:**
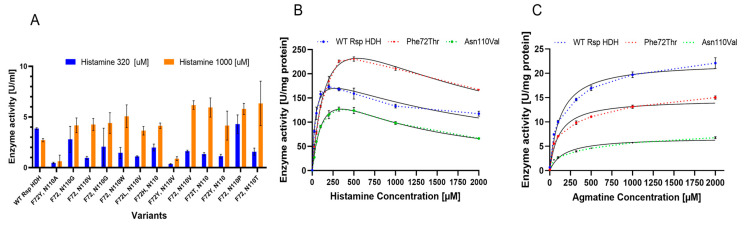
(**A**) Histamine activity at 320 µM (blue) and 1000 µM (orange) of selected variants from the double SSM library. This screen parameter was included to assess the substrate inhibition effect in the generated HDH variants. F72 and N110 mean no amino acid substitutions at these positions. (**B**) Substrate saturation assays to estimate kinetic parameters of WT Rsp HDH and variants Phe72Thr and Asn110Val. Enzymatic activity reactions using histamine (**B**) and agmatine (**C**) were performed by measuring the initial reaction rate (linear range) in microtiter plates as described above at a concentration of 25, 50, 100, 200, 320, 500, 1000, and 2000 µM substrate. Initial rates were calculated as µmol of released WST-8 formazan per mg of enzyme per minute. The obtained initial rate values for each substrate concentration were used for Substrate Inhibitor (histamine) and Michaelis–Menten (agmatine) fittings (black) using Graph Pad. In addition, the *V_max_*, *K_m_*, and *K_i_* values obtained from these fittings were used as reference values for analyzing the catalytic behavior of both enzyme variants. All measurements were performed in triplicate.

The screening revealed several variants with increased histamine/agmatine ratios; however, those with higher activity ratios only showed amino acid substitutions at positions 72 or 110 (Figure 4A). Substitutions appearing more often for position 72 among rescreened variants were Trp, Tyr, and His, and Leu, Val, Thr, and Ser were found less frequently. On the other hand, for position 110, Val, Trp, Gly, and Thr were found more often, whereas Leu, Pro, Arg, and His were also found. Interestingly, Trp, with only one codon in thirty-two available, was seen several times in rescreened variants at either position 72 or 110, strongly suggesting that this bulky hydrophobic residue plays a role in the substrate binding or the reaction mechanism; however, no variant with Trp simultaneously at positions 72 and 110 was identified, whereas Tyr72 was found in combination with other amino acid substitutions for variants selected for decreased substrate inhibition.

When histamine/agmatine (substrate specificity) and histamine 1000 µM/histamine 320 µM (substrate inhibition) activity ratios were considered, it could be proposed that positions 72 and 110 affect both substrate specificity and substrate inhibition. 

From the identified variants, two were selected for further evaluation, Phe72Thr and Asn110Val, which also showed good histamine/agmatine ratio performance. Interestingly, for both screening parameters, no variants with double substitutions showed higher performance than the single-substituted Phe72Thr and Asn110Val variants, probably related to low compatibility for amino acid combinations at positions 72 and 110 or a selection bias resulting from applying multiple selection criteria during screening, such as thermal resistance.

Variants Phe72Thr and Asn110Val were purified using immobilized metal affinity chromatography (IMAC) and compared regarding kinetic parameters using histamine and agmatine as substrates (Figure 4B,C). Enzymatic activity for histamine and agmatine was measured via the initial reaction rate (linear range) in microtiter plates as described in Materials and Methods, at substrate concentrations from 0 to 2000 µM. As previously described for this enzyme, a clear substrate inhibition curve for histamine was observed for all variants [9]. Therefore, the substrate saturation was adjusted to the substrate inhibition equation (*V* = *V_max_*×[S]/(*K_m_* + X×(1 + [S]/*K_i_*))) (Figure 4B). On the other hand, for agmatine, a Michaelis–Menten fitting (*V* = (*V_max_*×[S]/(*K_m_* + [S])) was used to describe the saturation curves (Figure 4C). *V_max_*, *K_m_*, and *K_i_* values obtained for these fittings were used as reference values to compare the catalytic behavior of both enzyme variants against WT Rsp HDH (Table 1).

For histamine, *V_max_* values increased for the variants compared with those for WT Rsp HDH, especially for variant Phe72Thr (398 compared with 216 µmol WST-8 formazan per mg enzyme per min). On the other hand, *K_m_* values increased for both variants, usually observed when screening was performed at substrate concentrations higher than *K_m_,* and variants showing higher activity were selected [28]. Thus, the *V_max_*/*K_m_* values for the variants were reduced for histamine compared with those for WT Rsp HDH. Finally, observed *K_i_* values for the variants decreased compared with those for WT Rsp HDH, meaning that the inhibition effect of the substrate should be observed at lower concentrations if the *V_max_* and *K_m_* values were the same as WT Rsp HDH. Variant Asn110Val showed a similar *V_max_* value to variant WT Rsp HDH. Still, the smaller *K_i_* value meant substrate inhibition was observed at lower concentrations for Asn110Val, thus hindering the observed activity at histamine concentrations higher than 250 µM.

For agmatine, an overall decrease in the specific activity was observed for the HDH variants. The *V_max_* value decreased for Asn110Val and Phe72Thr compared with that for WT Rsp HDH. On the other hand, the *K_m_* value did not change dramatically and was reduced for variant Phe72Thr to 112.9 µM, whereas for variant Asn110Val, it was increased to 170.1 µM. Consequently, the *V_max_*/*K_m_* values were reduced compared with those for WT Rsp HDH.

Overall, variant Phe72Thr showed an increased activity against histamine and a decreased activity against agmatine when compared with WT Rsp HDH; however, activity at lower substrate concentrations was decreased for the variant, explained by the increased *K_m_* value of 194.6 µM, compared with the original 38.24 µM of the WT Rsp HDH. Therefore, selecting enzyme variants with higher *V_max_* and higher *K_m_* is common when screening using substrate concentrations above *K_m_*. On the other hand, when screening using substrate concentrations under *K_m_*, there is a risk of selecting variants with lower activity at higher concentrations, and in this case, also with lower *K_i_* values, meaning a higher substrate inhibition at lower histamine concentrations.

**Figure 5:**
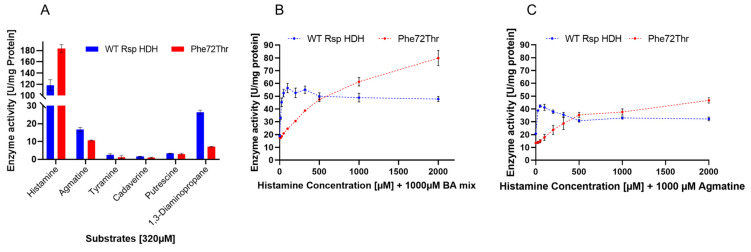
(**A**) Activity profile of purified WT Rsp HDH and variant Phe72Thr against histamine and other biogenic amines measured at 320 µM for each substrate. It can be observed that the histamine/agmatine and histamine/1,3-diaminopropane activity ratios are increased for the Phe72Thr variant. (**B**) Activity measurements using histamine saturation curves were performed for WT Rsp HDH and variant Phe72Thr in the presence of a biogenic amine mixture (“BA mix”) (**B**) containing agmatine, tyramine, cadaverine, putrescine, and 1,3-diaminopropane (200 µM each, 1000 µM total. (**C**) Activity measurements using histamine saturation curves were performed for WT Rsp HDH and variant Phe72Thr in the presence of 1000 µM agmatine.

The activity profile of purified WT Rsp HDH and variant Phe72Thr against histamine and other biogenic amine was measured at 320 µM for each substrate (Figure 5A), evidencing a desired decrease in calculated specific activity of variant Phe72Thr for agmatine and 1,3-diaminopropane, both substrates reported as the main secondary substrates for *Rhizobium* sp. HDH [9,11]. Interestingly, the selected variant showed a sharper decrease in activity for 1,3-diaminopropane, a substrate not used in the screening process since agmatine was reported as a common alternative substrate for both HDHs [9,17]. However, this decrease in activity suggests that activity against agmatine and 1,3-diaminopropane could co-evolve in screening events. Additionally, this indicates that substrate recognition for the amine moiety is probably similar to that for agmatine and 1,3-diaminopropane. Therefore, this work shows the observed correlation for these molecules as interferents for HDH and in the observed activity profile of the variant Phe72Thr.

Additionally, activity measurements using histamine saturation curves were performed for WT Rsp HDH and variant Phe72Thr in the presence of a biogenic amine mixture (“BA mix”) containing agmatine, tyramine, cadaverine, putrescine, and 1,3-diaminopropane (200 µM each, 1000 µM total, (Figure 5B), or in the presence of 1000 µM agmatine, (Figure 5C)) to observe the interfering effect of these molecules on HDH activity, simulating complex samples for histamine quantification. In both cases, a decrease in activity was observed for the HDH variant at the lowest substrate range; however, the shape of the enzyme activity curves was very different for Wt Rs HDH and Phe72Thr. The former, in the presence of both 1000 µM BA mix and 1000 µM agmatine, showed maximum activity at around 100 µM histamine, maintaining and decreasing the observed activity at higher histamine concentration, similar to the substrate inhibition curve observed in Figure 5B. On the other hand, the activity of variant Phe72Thr increased with higher histamine concentration, both in the presence of 1000 µM BA mix and 1000 µM agmatine, up to 2000 µM histamine.

For Phe72Thr, instead of substrate inhibition, a substrate saturation behavior was observed when other biogenic amines were present in the reaction, contrary to the behavior observed for the pure histamine activity curve. This change in activity versus substrate concentration response could be useful for histamine quantification applications, since it could be modeled to relate enzyme activity and histamine concentration for a wider substrate range (up to 2000 µM histamine) than WT Rs HDH, which, despite having a higher sensitivity, peaks at 100 µM histamine. Furthermore, this change in activity profile also suggests that position Phe72 could be involved not only in substrate specificity but also in substrate inhibition in Rsp HDH, as suggested by other authors for *N. simplex* HDH. There could be a close relationship between catalysis rate and substrate inhibition, where a decrease in substrate inhibition is linked to an increased K_m_ for histamine [21], which was also observed in this work for the Phe72Thr variant, compared with WT Rsp HDH. 

**Figure 6:**
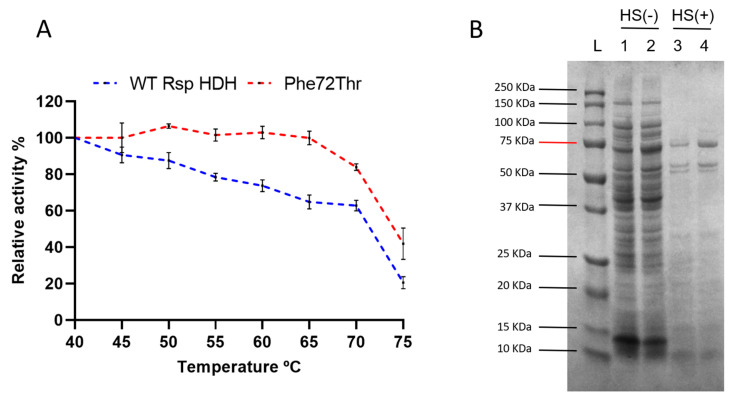
Temperature resistance of WT Rsp HDH compared with that of variant Phe72Thr. (**A**) Residual activity of purified WT Rsp HDH and variant Phe72Thr. The purified enzyme was incubated at different temperatures for 20 min and then cooled to 8 °C. Subsequently, enzyme activity was measured at 37 °C. (**B**) SDS-PAGE analysis of cell lysis supernatant containing recombinant Rsp HDH before (HS(−)) and after (HS(+)) heat shock (60 °C for 20 min) and centrifugation. After heat shock, a main protein band remains soluble around the 75 kDa migration distance. The Rsp HDH construct has a theoretical molecular weight of 78.91 kDa, corresponding to the observed remaining soluble band. In addition, two protein bands remain soluble at around 60 kDa. L = Molecular weight standard, 1 = WT Rsp HDH soluble lysate; 2 = Phe72Thr soluble lysate; 3 WT Rsp HDH soluble lysate after incubation at 60 °C for 20 min; 4 = Phe72Thr soluble lysate after incubation at 60 °C for 20 min.

Finally, a thermal resistance profile was performed to assess the residual activity of WT Rsp HDH and the variant Phe72Thr after incubation at different temperatures (Figure 6A). Variant Phe72Thr was expected to be at least as thermally resistant as WT Rsp HDH since a heat shock step was included during the screening process for the simultaneous SSM libraries. Variant Phe72Thr showed an increase in thermal resistance, with no activity loss after 20 min incubation at 65 °C, whereas WT Rsp HDH kept ~70% of its initial activity after the same process. After incubation at 75 °C, variant Phe72Thr maintained ~40% of the initial activity; in contrast, WT Rsp HDH showed ~20% of the initial activity. Figure 6B shows that after heat shock treatment, recombinant WT Rsp HDH and the Phe72Thr variant remain soluble (lanes 3 and 4).

In contrast, most other proteins present in *E. coli* lysate were not found in the supernatant after centrifugation. This increase in thermal resistance may be due to activity/stability compensation where the observed reduced affinity for the substrate could increase the interaction with water, thus increasing the overall stability of the enzyme [29,30]. By improving its initial thermal resistance, variant Phe72Thr is an interesting candidate for further enzyme processing, such as quick purification via thermal unfolding or immobilization in solid supports for quantitative color-based (strip type) histamine detection applications. 

**Figure 7:**
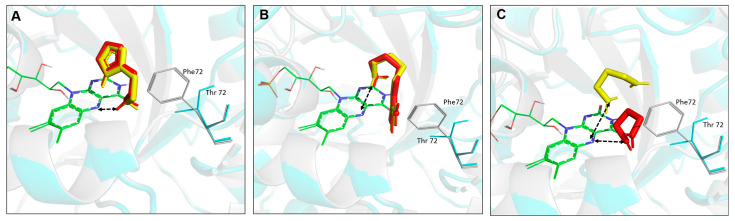
Molecular docking of substrates to *Rhizobium* sp. HDH. (**A**): Histamine; (**B**): agmatine; (**C**): 1,3-Diaminopropane. WT Rsp HDH is grey, and the HDH Phe72Thr variant is cyan. Substrates in WT Rsp HDH are shown in red and those in variant Phe72Thr in yellow. Reciprocal dashed arrows show the distance between the N5 atom of the FAD and the Nitrogen atom (hydride donor) of substrates.

Computational analysis was conducted to elucidate structure–function relationships on generated Rsp HDH variants regarding potential interaction with histamine, agmatine, and 1,3-diaminopropane. First, the Phe72Thr substitution was introduced using FoldX Suite [31,32], and energy was minimized. Then, independently, WT Rsp HDH and the Phe72Thr variant were used as receptors for molecular docking of histamine (Figure 7A), agmatine (Figure 7B), and 1,3-diaminopropane (Figure 7C). As stated above, according to the catalytic mechanism proposed for HDHs [22,23], the distance between the N5 atom of FMN and the N atom of the amino group in the substrate was used to identify catalytically competent binding modes. The closest distance for histamine in WT Rsp HDH was calculated to be 2.655 Å, with −4.37 kcal/mol binding energy.

In contrast, the Phe72Thr variant had a 2.679 Å distance and −4.39 kcal/mol binding energy, slightly lower than the value for WT Rsp HDH. Furthermore, the two other tested substrates had a longer distance than histamine, which correlates well with experimental results in which the WT enzyme had a higher affinity for histamine. In addition, the Phe72Thr variant showed a longer distance for agmatine and 1,3-diaminopropane compared with that for WT Rps HDH, which could explain the lower activity of the Phe72Thr variant towards these substrates compared with the original enzyme.

## 3. Materials and Methods

### 3.1. Reagents and Chemicals

Reagents and substrates used were of analytical grade or higher and purchased from Sigma-Aldrich (St. Louis, MO, USA), Merck (Kenilworth, NJ, USA), and Cayman Chemical (Ann Arbor, MI, USA). DNA ladder, enzymes, and dNTPs were purchased from New England Biolabs (Ipswich, MA, USA). Plasmid synthesis was performed by Biomatik (ON, Canada), oligonucleotide primers were used in mutagenesis, and DNA sequencing of variants was performed by Macrogen Inc. (Seoul, Republic of Korea). The DNA concentration was quantified in Biophotometer D30, using the µCuvette accessory (Eppendorf, Hamburg, Germany). 

### 3.2. Molecular Docking

The X-ray crystal homodimer structure of HDH WT was taken from the PDB databank (PDB ID: 6DE6, resolution 2.1 Å). Monomer A of the *Rhizobium* sp. HDH structure was used for in silico analysis since the active site was not in the interface of the homodimer structure. In silico generation of variants was performed using FoldX Suite [31] in YASARA Structure version 22.9.24 [20] and YASARA Plugin for FoldX [33]. Structures of HDH WT and generated variants were energy minimized with the steepest decent before molecular docking. Autodock was used for the molecular docking of substrates (histamine, agmatine, and 1,3-diaminopropane) within YASARA Structure [20]. One hundred Autodock runs were performed with a simulation cell boundary of 7.0 Å with true walls around the N5 atom of FMN. The default parameters within the YASARA dock_run macro file were used with the fixed backbone. The docking results were clustered by applying an RMSD cut-off of 0.7 Å. 

Most FAD-dependent dehydrogenases prefer hydride transfer, and flavins can accept hydride at N5 of the flavin from several organic substances [34,35]. Therefore, in our mechanism-based docking of substrates, based on the proposed mechanism for flavin-containing enzymes (Figure S6), the distance between the N5 atom of the FMN and the nitrogen atom (hydride donor) of substrates was applied as the catalytic distance criteria (Figure S1). Based on this, the molecular docking poses were analyzed by considering the orientation of the substrate’s aromatic ring towards the 6-S-CysFMN inside the binding pocket and the distance between the N5 atom of FMN and the N atom of the amino group in the substrate.

### 3.3. Plasmid Construction

The Histamine Dehydrogenase amino acid sequence, including a C-terminal 6x histidine tag, was reverse-translated into an ORF optimized for *E. coli* expression, and the gene was synthesized (Figure S7). The gene was then cloned into pET28a (+) using NdeI and EcoRI, resulting in pET28a-HDH for recombinant production of HDH. The resulting amino acid sequence is detailed in Figure S8.

### 3.4. Construction of Site Saturation Mutagenesis (SSM) Libraries for Rsp HDH

For each selected amino acid site, site saturation mutagenesis (SSM) libraries were constructed using a modified QuikChange PCR protocol, with pET28a-HDH as a template and single-stranded DNA oligonucleotide primers designed with NNK codon diversity (Table S4). The PCR reaction mix included Buffer Phusion 5X, 10 mM dNTP, 10 μM of each primer, 40 ng template, and Phusion High-Fidelity Polymerase in a total volume of 50 μL. For each SSM library, PCR was carried out in two steps; in the first one, the mix was generated with each corresponding Forward (Fwd) and Reverse (Rev) primers separately, and the following program was used: 95 °C for 30 s, 5 cycles; 98 °C for 10 s; 55 °C for 15 s; 72 ° C for 220 s; and finally 72 °C for 10 min. In the second step, the Forward and Reverse mixes were combined, and the PCR reaction was allowed to continue using the PCR program: 95 °C for 30 s, 20 cycles; 98 °C for 10 s; 55 °C for 15 s; 72 °C for 220 s; and finally 72 °C for 10 min. Next, the PCR product was treated with Dpnl (20 U, 37 °C, 18 h) to digest methylated template DNA. Dpnl was then inactivated (80 °C, 20 min). Finally, the resulting SSM libraries were transformed into chemically competent *E. coli* BL21 gold (DE3) using a standard heat shock transformation protocol. 

### 3.5. Cultivation and Recombinant Production of HDH-Variants in 96-Well Microtiter Plates

For each site saturation mutagenesis library, at least 90 *E. coli* BL21 gold (DE3) colonies transformed with the QuikChange PCR products from the libraries were picked and resuspended into 96-well flat bottom microtiter plates containing 150 µL of 1× LB (10 g/L tryptone, 5 g/L yeast extract, 10 g/L NaCl, 0.1% *v*/*v* glucose) + 50 µg/mL kanamycin, and incubated at 37 °C, 500 rpm for 15 h for master plate construction. In each plate, three wells with “wild type” HDH (WT Rsp HDH) and three without inoculum were left as positive and negative controls. Master plates were stored at −80 °C after adding and homogenizing each well with 80 µL of 50% *v*/*v* glycerol.

Recombinant production of the HDH libraries was carried out by replicating master plates into 96-well V-bottom microtiter plates, with a 96-well microtiter plate replicator containing 1 mL of 2 × LB (20 g L^−1^ tryptone, 10 g L^−1^ yeast extract, 10 g L^−1^ NaCl) + 50 µg mL^−1^ kanamycin, and incubated at 37 °C, 500 rpm for 8 h to an OD_600_ of 1. The turbidimetry corroborated a microtiter plate reader (Infinite M Nano plus, Tecan, Switzerland). HDH production was induced by adding 50 µL of IPTG to a final concentration of 0.3 mM in each well. A total of 15 h post-induction, microtiter plates were centrifuged at 4000 rpm, 15 °C for 20 min (Eppendorf 5810R), the supernatant was discarded, and the pellet was resuspended in 200 µL of 1 mg mL^−1^ lysozyme solution dissolved in lysozyme buffer (20 mM Tris-HCl pH 8, 2 mM EDTA, 1% TRITON X-100) and incubated for 1 h at 37 °C and 500 rpm for cell disruption. Finally, the supernatant was collected after centrifugation of the plates at 3220 g, 4 °C for 20 min, and transferred to a new 96-well flat bottom microtiter plate for enzyme activity measurements. The background activity of HDH from the Tris compound in the lysis buffer was measured and found to be less than 0.0001 AU/min). 

### 3.6. Enzyme Activity Assay for HDH and High-Throughput Screening of Libraries in 96-Well Microtiter Plates

The reaction mechanism was based on the decomposition of histamine through the action of histamine dehydrogenase in the presence of an electron carrier 1-methoxy-5-methylphenazinium methylsulfate (1-methoxy PMS), producing reduced 1-methoxy PMS, which transfers electrons to a chromogenic salt (2-(2-methoxy-4-nitrophenyl)-3-(4-nitrophenyl)-5-(2,4-disulfophenyl)-2H-tetrazolium monosodium salt (WST-8)), and the increase in absorbance over time can be detected at 460 nm. Therefore, the enzymatic activity of HDH was determined using the initial rate of formation of reduced WST-8 (formazan) up to 1 h at 37 °C via measuring the absorbance increase at 460 nm in a microtiter plate reader (Infinite M Nano plus, Tecan, Switzerland). The reaction was performed by adding 170 µL of buffer-substrate solution (83 mM glycine-NaOH buffer pH 9.0, 320 µM histamine dihydrochloride as substrate, 10 µM 1-methoxy PMS, and 100 µM WST-8) to the 96-well flat bottom microtiter containing 30 µL of the enzyme from the cell lysate containing recombinantly produced Rsp HDH. One international unit of activity was defined as the amount of HDH that produced 1 µmol of WST-8 formazan per minute under the assay conditions, considering a molar extinction coefficient of 0.036 µM^−1^ cm^−1^ [9]. Stock MTPs included 3 wells with unmutated Rsp HDH variants (WT Rsp HDH) and 3 wells containing no *E. coli* inoculation as internal controls. MTPs were grown overnight, sterile glycerol was added for cryopreservation, and the mixed plates were stored at −80 °C. For screening, plates were thawed and replicated in V-bottom expression plates, where recombinant Rsp HDH expression and cell lysis were performed. High-throughput screening of the enzymatic activity of the libraries was carried out with the following biogenic amines at a concentration of 320 µM as substrate: histamine dihydrochloride, 1,3-diamino propane, putrescine (1,4-diaminobutane), agmatine sulfate salt, tyramine (4-(2-aminoethyl)phenol), benzylamine, and cadaverine dihydrochloride. The enzymatic activity of the variants was compared with the different substrates. After SSM screening, twenty-six variants were selected for plasmid DNA sequence and analysis (Macrogen Inc., Seoul, Republic of Korea) to identify mutations in the Rsp HDH gene.

### 3.7. Characterization of HDH Enzyme Variants

For the characterization of the selected Rsp HDH enzyme mutants, the enzyme activity assay described above was used [8]. Six different substrates were analyzed, histamine dihydrochloride, 1,3-diamino propane, putrescine (1,4-diaminobutane), agmatine sulfate salt, tyramine (4-(2-aminoethyl)phenol), benzylamine, and cadaverine dihydrochloride, in concentrations ranging from 0 to 5000 µM dissolved in glycine buffer (100 mM, pH 9.0). 

### 3.8. Construction of Simultaneous Saturation Site-Directed Mutagenesis Libraries and Screening

A simultaneous saturation site library was constructed by combining the amino acid positions 72 and 110 of the selected Rsp HDH variants. The PCR conditions for a single SSM from the first round were used sequentially for positions 72 and 110. After transformation of the DpnI-digested PCR products, the recombinant enzyme was produced as described above, obtaining a cell lysate which was transferred to a 96-well PCR plate for an additional thermal resistance screening step, subjecting the enzyme-containing lysates to 60 °C for 20 min in a thermocycler (Esco Aeris, Esco, Horsham, PA, USA) where its enzymatic activity was subsequently measured as described above. Histamine 320 and 1000 µM and agmatine 1000 µM were used as substrates to measure enzyme activity before and after the thermal challenge. In addition, the selection of HDH variants was analyzed by those that showed an increase in the histamine/agmatine activity ratio.

### 3.9. Enzyme Purification

The HDH enzyme was purified using immobilized metal affinity chromatography (IMAC)—due to the histidine tail genetically added to the C-terminal of the enzyme—using a Ni Sepharose^TM^ 6 fast Flow column on the AKTAprime plus (GE, Chicago, IL, USA) FPLC system. The column (approx. 15 mL) was loaded with ten volumes of 100 mM nickel sulfate (NiSO_4_) and washed with five volumes of ultra-purified water. Before sample loading, the column was equilibrated with ten volumes of binding buffer (20 mM sodium phosphate, 500 mM sodium chloride, 10 mM imidazole, pH 7.4). The cell culture lysate was diluted 1:3 (*v*/*v*) in a binding buffer. Proteins bound to the column were eluted using elution buffer (20 mM sodium phosphate, 500 mM sodium chloride, 500 mM imidazole, pH 7.4) in a 0–50–100% gradient at a flow rate of 3 mL min^−1^, collecting 3 mL purified enzyme fractions. The fractions were measured for enzyme activity via the assay described above, using 320 µM histamine as substrate; those fractions that showed the highest enzymatic activity were pooled and concentrated in vivaspin^TM^ ultrafiltration columns (10K MWCO) using lysis buffer (Tris-HCl 20 Mm, EDTA 2 mM, Triton X-100 1%) centrifuged at 3220× *g* for 10 min to recover 2 to 3 mL of the enzyme. The samples were stored at 4 °C until use.

The purity of the HDH enzyme was confirmed using sodium dodecyl sulfate-polyacrylamide gel electrophoresis (SDS-PAGE), and enzyme concentration was estimated using IMAGE J software using a BSA standard curve (Figure S9).

### 3.10. Characterization of Purified Fractions

The purified enzymes were characterized using histamine and agmatine substrates in a range of concentrations from 25 to 2000 µM. The substrates were used separately and as a mixture to observe substrate interference. In addition, the temperature resistance of the purified variants was evaluated in a range of 40 to 75 °C. The purified enzyme was incubated for 20 min at different temperatures using a gradient Thermal Cycler, brought to room temperature, and its activity was subsequently measured at 37 °C. In addition, enzymatic activity was measured against the following substrates: histamine dihydrochloride, 1,3-diamino propane, putrescine, agmatine sulfate salt, tyramine, and cadaverine dihydrochloride. 

### 3.11. Sequencing Data Analysis

Clone Manager 9 Professional Edition Software (Scientific & Educational Software, Cary, NC, USA) was used for sequence analysis of mutations in HDH.

## 4. Conclusions

In this work, we proposed a protein engineering strategy for modulating the substrate specificity of *Rhizobium* sp. Histamine Dehydrogenase based on structural analysis and substrate docking. We found that positions Tyr171, Phe176, Trp263, Asp265, and Asp354 were highly sensitive to amino acid substitutions, suggesting a critical role for catalysis. On the other hand, libraries at positions Phe72 and Asn110 contained populations with variants having activity values close to the WT Rsp HDH, and selected variants came from these two libraries.

**Figure 8:**
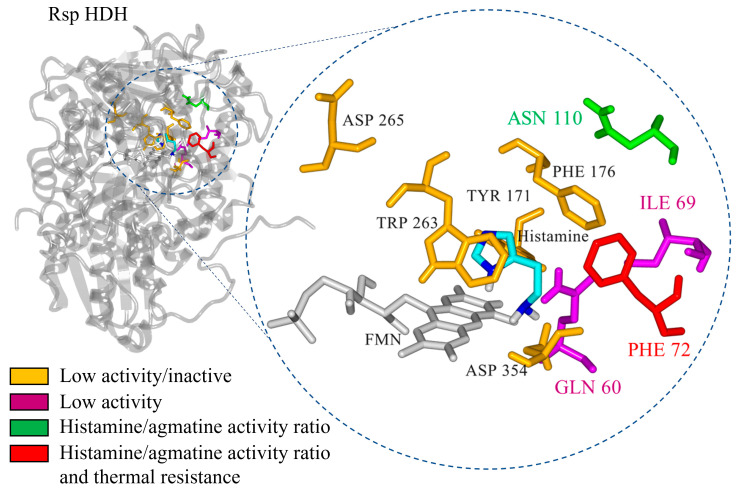
Summary of the functional effect of amino acid substitutions identified in this work for *Rhizobium* sp. HDH.

WT Rsp HDH and the identified Phe72Thr variant showed a clear substrate inhibition curve for histamine. In addition, an increased *V_max_* value was observed for the variant at the cost of an increased *K_m_*. On the other hand, the overall activity against the second substrate agmatine decreased for the identified variants, suggesting the role of positions 72 and 110 in substrate specificity in *Rhizobium* sp. HDH (Figure 8). 

A decreased activity against 1,3-diaminopropane further reinforced this position’s role in substrate specificity [9,11]. Furthermore, structural protein modeling and substrate docking studies revealed that the introduced amino acid substitution affected the most likely poses for agmatine and 1,3-diaminopropane, increasing distances between catalytic atoms and correlating with the experimental results, which would help to propose amino acid substitution to modulate substrate specificity further.

Variant Phe72Thr had lower *V_max_*/*K_m_* values for histamine compared with those for WT Rsp HDH. However, at higher histamine concentrations (Over 250 µM, ~28 ppm histamine), variant Phe72Thr showed higher activity, peaking at 500 µM (~56 ppm) histamine. In the presence of other biogenic amines, WT Rsp HDH activity peaked at 100 µM histamine, whereas the activity of variant Phe72Thr increased proportionally to histamine concentration up to 2000 µM (~224 ppm), a feature that is potentially helpful for histamine detection and elimination from food matrices with high biogenic amine content, such as fermented products. The increased *K_m_* value, decreased substrate inhibition, and biogenic amine interference observed for variant Phe72Thr support a tradeoff between substrate affinity and substrate inhibition in the catalytic mechanism of HDHs [21]. Considering this tradeoff for future enzyme engineering of HDH could lead to breakthroughs in performance increase and understanding of this enzyme class.

Finally, we observed a higher residual activity after incubation from 40 to 75 °C for the variant Phe72Thr, remaining soluble at temperatures where most *E. coli* proteins sediment (Figure 6B), making it appropriate for technological applications and processes such as enzyme immobilization and Immobilized Biocatalyst Engineering (IBE) [36].

Further research on the performance of Rsp HDH, its variants, and other novel hydrogenase backbones is needed to determine the suitability for food histamine detection and degradation and to obtain more insights into the complex substrate inhibition and substrate specificity mechanisms of this enzyme family.

## Figures and Tables

**Table 1:** Observed kinetic parameters for purified WT Rsp HDH and variants Phe72Thr and Asn110Val using histamine and agmatine as substrates.

	Histamine (Substrate Inhibition)				Agmatine (Michaelis–Menten)		
Variant	Observed *V_max_* *	Observed *K_m_* (µM)	*V_max_*/*K_m_*	Observed *K_i_* (µM)	Observed *V_max_* *	Observed *K_m_* (µM)	*V_max_*/*K_m_*
WT Rsp HDH	216.3	38.24	5.66	2059.0	22.31	130.1	0.17
Phe72Thr	398.2	194.6	2.04	1514.0	14.65	112.9	0.13
Asn110Val	242.5	159.5	1.52	771.9	6.77	170.1	0.04

* (µmol WST-8 formazan per mg enzyme per min).

## Data Availability

The corresponding author can make any data or material supporting this study’s findings available upon request.

## Supplementary Materials

The following supporting information can be downloaded at: https://www.mdpi.com/article/10.3390/molecules28093748/s1, Figure S1: Catalytically important distance criteria based on the proposed catalytic mechanism of HDH. Table S1: Substrates along with their docking binding energy towards Rsp HDH WT and F72T variant in kcal/mol. Figure S2: Cavity analysis of HDH using the Caver Web tool 1.0. Table S2: List of residues involved in predicted active cavity identified by the Caver Web tool 1.0. Figure S3: Tunnels analysis in HDH using CaverWeb Tool. Table S3: Tunnels analysis in HDH using CaverWeb Tool. Table S4: Site Saturation Mutagenesis PCR primers used to construct mutant Rsp HDH libraries. Table S5: Evolutionary conservation analysis of selected residues of HDH via ConSurf and UET servers. Figure S4: Evolutionary conservation analysis of HDH using Consurf. Figure S5: Evolutionary Trace Analysis of HDH using UETserver. Figure S6: Proposed catalytic mechanism of HDHs. Figure S7: DNA sequence used in this work for *E. coli* recombinant production of *Rhizobium* Sp. HDHs. Figure S8: Amino acid sequence used in this work for *Rhizobium* Sp. HDHs. Figure S9: SDS-PAGE analysis of purified HDH WT Rsp.

## Sample Availability Statement

Samples are available from the authors.
