# Supplementary material for: Modulating Substrate Specificity of Rhizobium sp. Histamine Dehydrogenase through Protein Engineering for Food Quality Applications
Source: Molecules. 2023 Apr 26;28(9):3748. doi: 10.3390/molecules28093748 (PMC10180351; doi:10.3390/molecules28093748)
Supplement: Supplementary file 1 [file molecules-28-03748-s001.zip › molecules-2354280-supplementary.pdf]

Supplementary Materials

Modulating substrate specificity of *Rhizobium sp.* Histamine Dehydrogenase through protein engineering for food quality applications.

Karen Rodríguez-Núñez[1], Alejandra Cortés-Monroy[1], Marcela Serey[1], Yunus Ensari[2], Mehdi D. Davari[3], Claudia Bernal[4], and Ronny Martinez[1*]

[1] Departamento de Ingeniería en Alimentos, Universidad de La Serena, Av. Raúl Bitrán 1305, 1720010 La Serena, Chile.
[2] Department of Bioengineering, Faculty of Engineering and Architecture, Kafkas University, Kars, Turkey.
[3] Department of Bioorganic Chemistry, Leibniz Institute of Plant Biochemistry, Weinberg 3, D-06120, Halle, Germany.
[4] Instituto de Investigación Multidisciplinaria en Ciencia y Tecnología, Universidad de La Serena, Av. Raúl Bitrán 1305, 1720010 La Serena, Chile.

***Correspondence to:**
Dr. Ronny Martínez
Departamento de Ingeniería en Alimentos, Universidad de La Serena, Av. Raúl Bitrán 1305, 1720010 La Serena, Chile
Tel.: +56 51 2334661
Fax: +56 51 2204446
E-mail: remartinez@userena.cl

Table of contents

**Figure/Table**	**Page**
**Figure S1: Catalytically important distance criteria based on the proposed catalytic mechanism of HDH**	**S3**
**Table S1: Substrates along with their docking binding energy towards Rsp HDH WT and F72T variant in kcal/mol**	**S3**
**Figure S2. Cavity analysis of HDH using the Caver Web tool 1.0**	**S4**
**Table S2: List of residues involved in predicted active cavity identified by the Caver Web tool 1.0**	**S4**
**Figure S3. Tunnels analysis in HDH using CaverWeb Tool**	**S5**
**Table S3. Tunnels analysis in HDH using CaverWeb Tool.**	**S6**
**Table S4: Site Saturation Mutagenesis PCR primers used to construct mutant Rsp HDH libraries**	**S6**
**Table S5: Evolutionary conservation analysis of selected residues of HDH via ConSurf and UET servers**	**S7**
**Figure S4. Evolutionary conservation analysis of HDH using Consurf**	**S8-S9**
**Figure S5. Evolutionary Trace Analysis of HDH using UETserver**	**S10**
**Figure S6. Proposed catalytic mechanism of HDHs**	**S11**
**Figure S7: DNA sequence used in this work for E. coli recombinant production of *Rhizobium Sp.* HDHs**	**S12**
**Figure S8: Amino acid sequence used in this work for *Rhizobium Sp*. HDHs**	**S13**
**Figure S9: SDS-PAGE analysis of purified HDH WT Rsp**	**S14**
**Supporting References**	**S15**

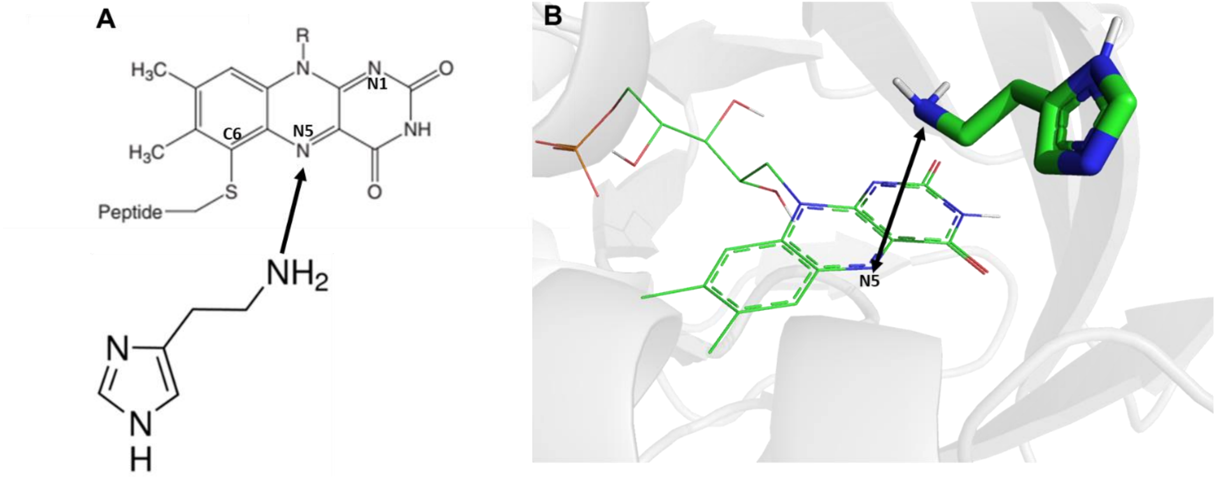

Figure S1: Catalytically important distance criteria based on the proposed catalytic mechanism of HDH [1, 2]. **A**) Schematic representation of hydride transfer from histamine to N5 atom of FAD (Figure was adapted from Tsutsumi *et al* [1] and Huang *et al.* [2]); **B**) Catalytically active pose obtained from docking of histamine to Rsp HDH. The reciprocal arrow indicates the catalytically important distance between histamine and FAD.

Table S1: Substrates along with their docking binding energy towards *Rsp* HDH WT and F72T variant in kcal/mol. Distance indicates the catalytically important distance (in Å) between the N5 atom of the FAD and the Nitrogen atom (hydride donor) of substrates.

Substrate	Variant	Distance [Å]	Binding energy [kcal/mol]
Histamine	WT	2.655	-4.37
	F72T	2.679	-4.39
Agmatine	WT	3.677	- 3.42
	F72T	3.702	-3.17
1,3-Diaminopropane	WT	3.114	-1.78
	F72T	3.571	-1.84

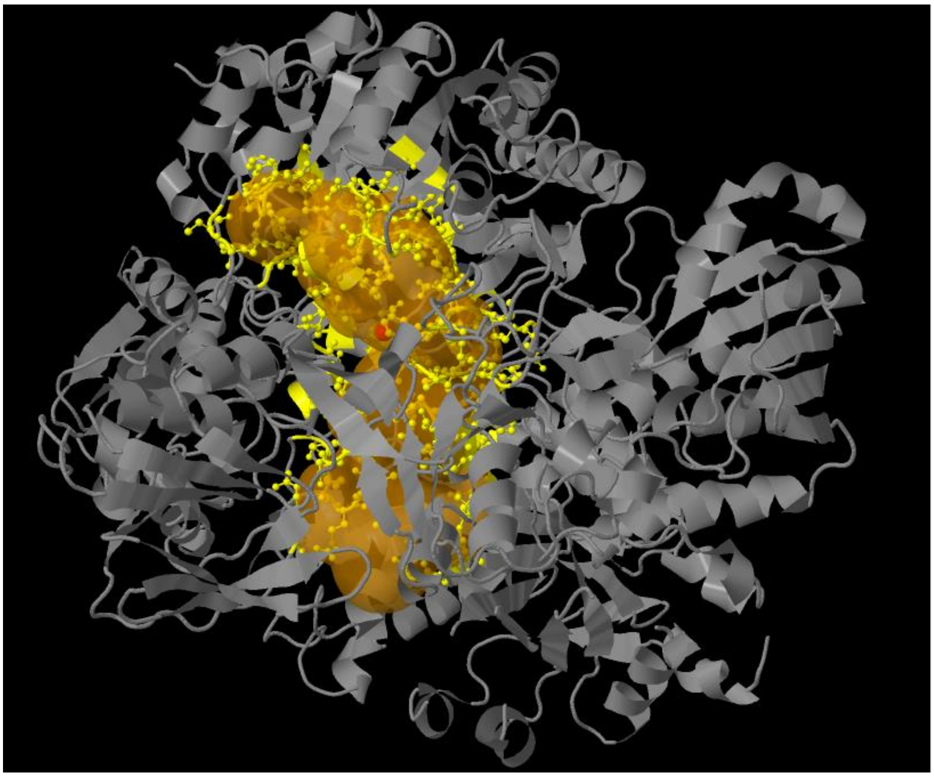

Figure S2. Cavity analysis of HDH using the Caver Web tool 1.0 [3]**.** Active cavity identified in the substrate-binding domain of the HDH (PDB ID: 6DE6). The cavity with 66 lining residues, pocket relevance score; 100 % , volume 4255 A^3, draggability: 0.63. Residues constitute the cavity are shown as yellow ball and stick

Table S2: List of residues involved in predicted active cavity identified by the Caver Web tool 1.0 [3]. Residues selected for SSM were shown as bold.

V27	**N110**	G175	**D265**	C319	F368
P28	P112	**F176**	C266	A320	R491
H29	F114	R225	S267	R321	W562
C30	I128	D229	G268	P322	R564
E59	R129	E230	K273	I324	N565
**Q60**	T130	T231	E274	C350	L567
**I69**	F131	I232	A277	I351	
T70	T132	Q260	Q278	**D354**	
P71	N133	G261	V296	M355	
**F72**	D134	T262	G297	T356	
E74	**Y171**	**W263**	R298	M357	
Q103	H174	E264	F299	S360	

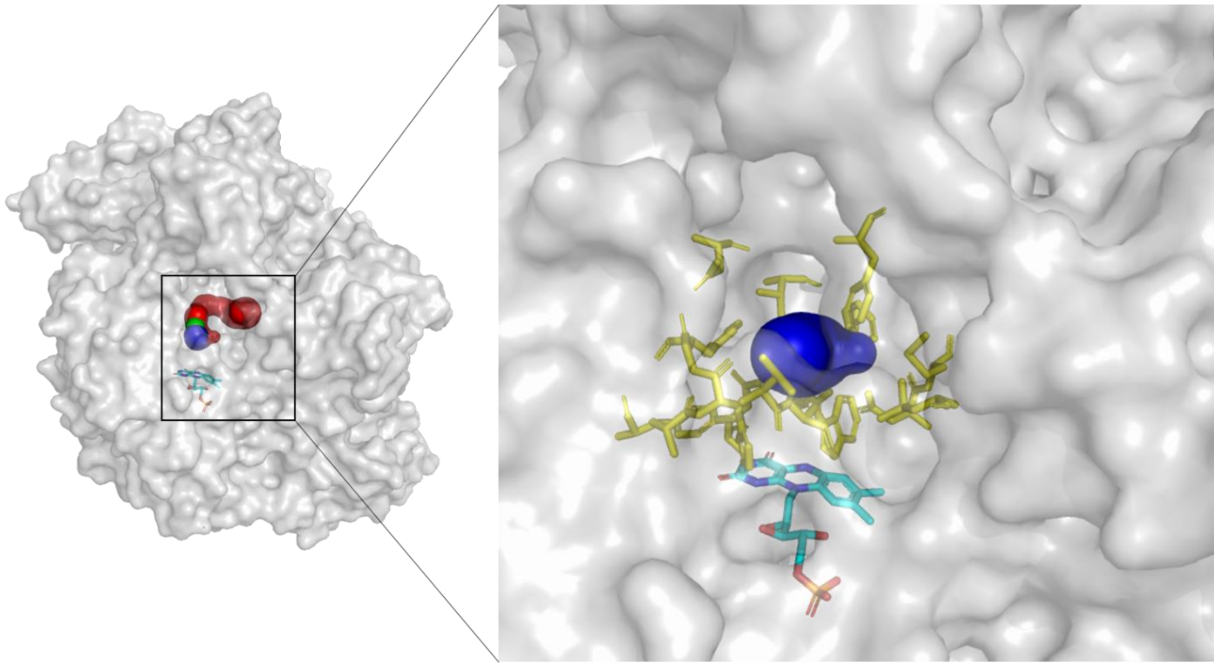

Figure S3. Tunnels analysis in HDH using CaverWeb Tool [3]. Left; tunnel 1, tunnel 2, and tunnel 3 was shown in blue, green and blue respectively. Right; Detailed representation of the residues involved in tunnel 1 identified by CaverWeb tool. Residues were shown as yellow stick and FAD was shown as element stick. Pymol was used for visualization and hydrophobic hydrogens were removed for better visualization.

Table S3. Tunnels analysis in HDH using CaverWeb Tool [3]. * color represents the tunnels shown in Figure S3

Tunnel	Bottleneck Radius [Å]	Length [Å]	Curvature	Throughput	Color*
1	1.3	6.2	1.4	0.84	Blue
2	1.3	8.7	1.9	0.77	Green
3	1.3	24.3	3.2	0.63	Red

Table S4: Site Saturation Mutagenesis PCR primers used to construct mutant Rsp HDH libraries. Degenerated NNK codons were used for amino acid diversity generation in the selected positions. In addition, the binding 5' and 3' sequences flanking the NNK codons were designed to have a 55°C melting temperature; thus, all primers can be run in a single PCR program. The degenerated codon for each primer is highlighted in bold letters.

Library	Target	Primer	Sequence
1	Gln60	Gln60sat_FW	GTT GGG GTG TTA TCT TCA CCG AA **NNK** ACC GAA ATG CAC CAC ACC
		Gln60sat_RV	GGT GTG GTG CAT TTC GGT **MNN** TTC GGT GAA GAT AAC ACC CCA AC
2	Ile69	Ile69sat_FW	GAA ATG CAC CAC ACC TCT GAA **NNK** ACC CCG TTC ATC GAA CTG
		Ile69sat_RV	CAG TTC GAT GAA CGG GGT **MNN** TTC AGA GGT GTG GTG CAT TTC
3	Phe72	Phe72sat_FW	CAC CTC TGA AAT CAC CCC G **NNK** ATC GAA CTG CGT CTG TGG
		Phe72sat_RV	CCA CAG ACG CAG TTC GAT **MNN** CGG GGT GAT TTC AGA GGT G
4	Asn110	Asn110sat_FW	GCT GGC GTA CTC TGG TAT C **NNK** GGT CCG AAC TTC TAC ACC AAA G
		Asn110sat_RV	CTT TGG TGT AGA AGT TCG GAC C**MN N**GA TAC CAG AGT ACG CCA GC
5	Tyr171	Tyr171sat_FW	GTT TCG ACC TGA TCT GCC TG **NNK** GGT GCG CAC GGT TTC
		Tyr171sat_RV	GAA ACC GTG CGC ACC **MNN** CAG GCA GAT CAG GTC GAA AC
6	Phe176	Phe176sat_FW	CGG TGC GCA CGG T **NNK** GGT ATC TTC CAG CAC TTC CTG TC
		Phe176sat_RV	GAC AGG AAG TGC TGG AAG ATA CC**M NN**A CCG TGC GCA CCG
7	Trp263	Trp263sat_FW	GGC GCA GGG GAC C **NNK** GAA GAC TGC TCT GGT CCG TC
		Trp263sat_RV	GAC GGA CCA GAG CAG TCT TC**M NN**G GTC CCC TGC GCC
8	Asp265	Asp265sat_FW	GCA GGG GAC CTG GGA A **NNK** TGC TCT GGT CCG TCT CG
		Asp265sat_RV	CGA GAC GGA CCA GAG CA**M NN**T TCC CAG GTC CCC TGC
9	Asp354	Asp354sat_FW	CAA CAT CTG CAT CAC CGG T **NNK** ATG ACC ATG TCT ATC TCT CGT TGC
		Asp354sat_RV	GCA ACG AGA GAT AGA CAT GGT CAT **MNN** ACC GGT GAT GCA GAT GTT G

Table S5: Evolutionary conservation analysis of selected residues of HDH via ConSurf [4] and UET servers [5].

**Residue**	**ConSurf**	**Evolutionary Trace Score (rvET)**
**Q60**	7	53.69
**I69**	6	54.78
**F72**	1	56.61
**E74**	7	57.24
**N110**	4	43.41
**F131**	1	122.91
**Y171**	9	20.79
**A173**	6	27.84
**H174**	9	20.05
**G175**	3	21.74
**F176**	7	12.71
**T262**	3	122.91
**W263**	9	122.91
**D265**	6	59.56
**D354**	9	48.01
**M355**	3	47.38
**L567**	6	61.06

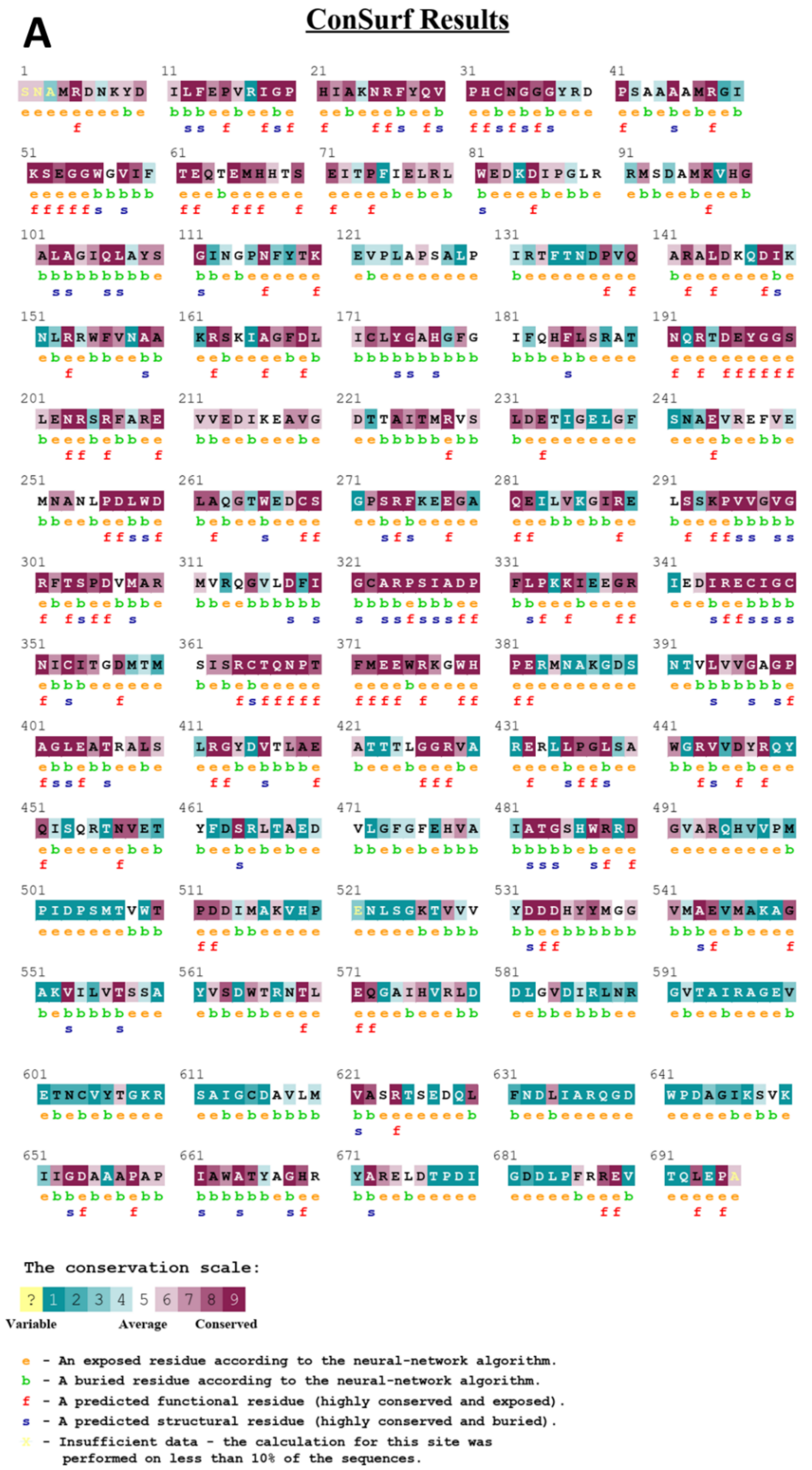
A
ConSurf Results
The conservation scale:
? 1 2 3 4 5 6 7 8 9
Variable
Average
Conserved
e - An exposed residue according to the neural-network algorithm.
b - A buried residue according to the neural-network algorithm.
f - A predicted functional residue (highly conserved and exposed).
s - A predicted structural residue (highly conserved and buried).
X - Insufficient data - the calculation for this site was performed on less than 10% of the sequences.

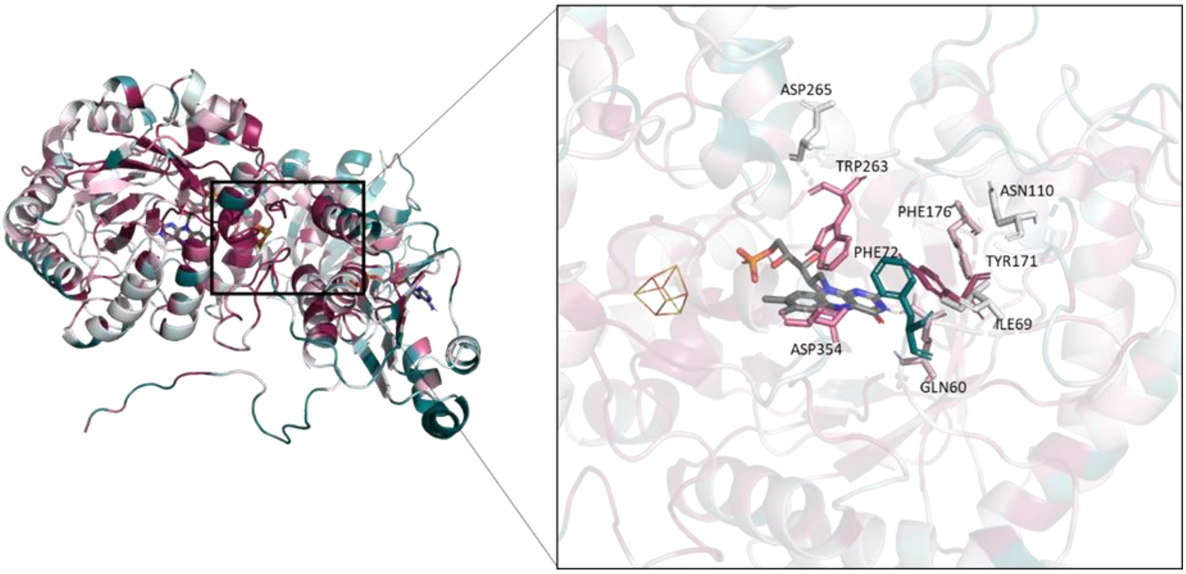

Figure S4. Evolutionary conservation analysis of HDH using Consurf [5]. A) Sequence **B)** Cartoon representation of 3D structure of the *Rsp* HDH. The residues are color-coded as indicated by the conservation scale.

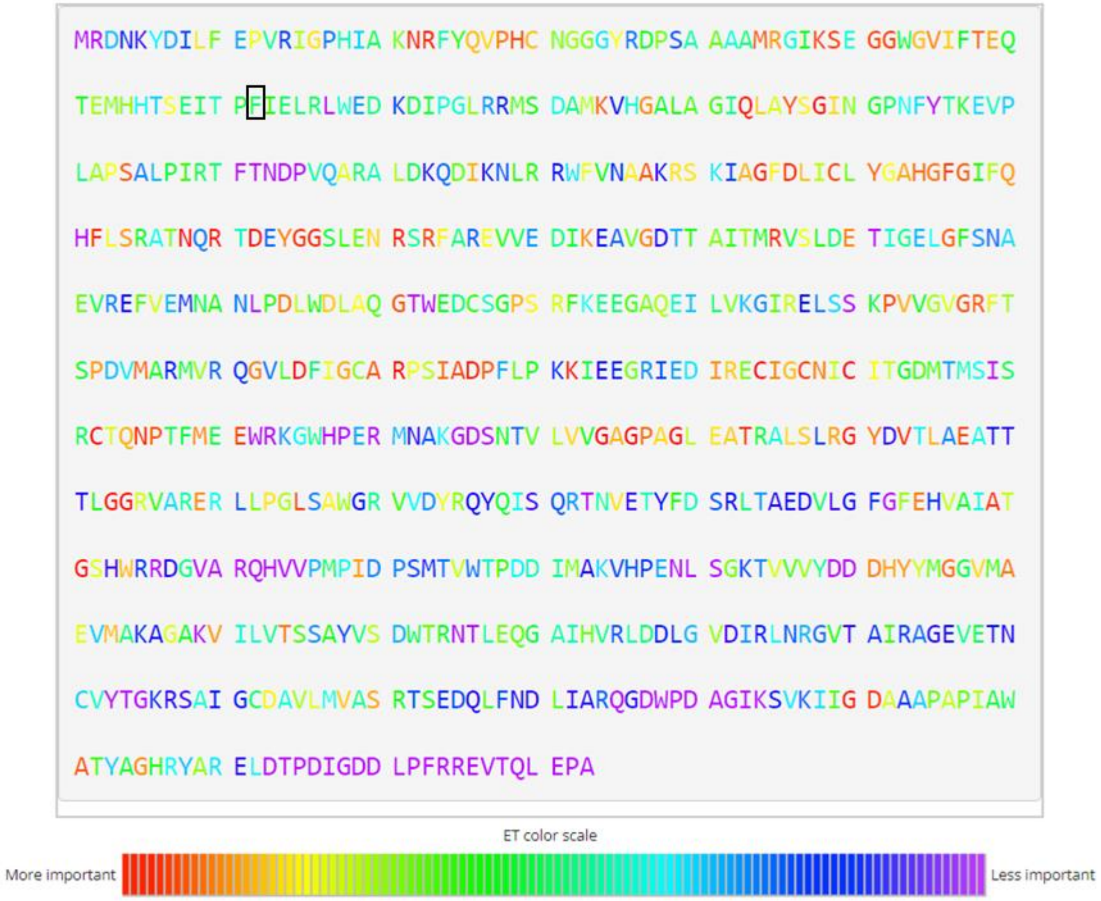

Figure S5. Evolutionary Trace Analysis of HDH using UETserver [5]. The evolutionary importance of residues is color coded from red (more important) to violet (less important). Residue Phe72 is marked in black.

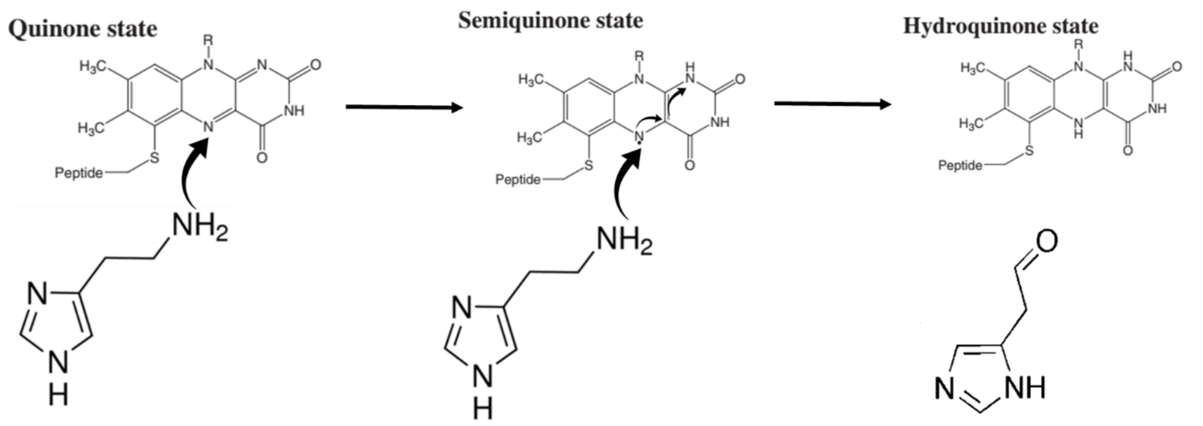

Figure S6. Proposed catalytic mechanism of HDHs. Flavin can undergo hydride transfer; Flavin is able to accept hydride at N5 of the flavin nucleus from NAD(P)H and several organic substances. Thus, hydrides at the amine group of histamine are transferred to N5 atom of FMN molecule. The first hydride is transferred to N5 and then is transferred to N1. Later, the second hydride is transferred to N5 and FMN turns to its hydroquinone state. Thus, the distance between amine group of histamine and N5 of FMN is important for the catalytic mechanism of HDH. Figure was adapted from Tsutsumi *et al* [1] and Huang *et al.* [2].

ATGGGCAGCAGCCATCATCATCATCATCACAGCAGCGGCCTGGTGCCGCGCGGCAGCCAT
ATGCGTGACAACAAATACGACATCCTGTTCGAACCGGTTCGTATCGGTCCGCACATCGCG
AAAAACCGTTTCTACCAGGTTCCGCACTGCAACGGTGGTGGTTACCGTGACCCGTCTGCG
GCGGCGGCGATGCGTGGTATCAAATCTGAAGGTGGTTGGGGTGTTATCTTCACCGAACAG
ACCGAAATGCACCACACCTCTGAAATCACCCCGTTCATCGAACTGCGTCTGTGGGAAGAC
AAAGACATCCCGGGTCTGCGTCGTATGTCTGACGCGATGAAAGTTCACGGTGCGCTGGCG
GGTATCCAGCTGGCGTACTCTGGTATCAACGGTCCGAACTTCTACACCAAAGAAGTTCCG
CTGGCGCCGTCTGCGCTGCCGATCCGTACCTTCACCAACGACCCGGTTCAGGCGCGTGCG
CTGGACAAACAGGACATCAAAAACCTGCGTCGTTGGTTCGTTAACGCGGCGAAACGTTCT
AAAATCGCGGGTTTCGACCTGATCTGCCTGTACGGTGCGCACGGTTTCGGTATCTTCCAG
CACTTCCTGTCTCGTGCGACCAACCAGCGTACCGACGAATACGGTGGTTCTCTGGAAAAC
CGTTCTCGTTTCGCGCGTGAAGTTGTTGAAGACATCAAAGAAGCGGTTGGTGACACCACC
GCGATCACCATGCGTGTTTCTCTGGACGAAACCATCGGTGAACTGGGTTTCTCTAACGCG
GAAGTTCGTGAGTTTGTTGAAATGAACGCGAACCTGCCGGACCTGTGGGACCTGGCGCAG
GGGACCTGGGAAGACTGCTCTGGTCCGTCTCGTTTCAAAGAAGAAGGTGCGCAGGAAATC
CTGGTTAAAGGTATCCGTGAACTGTCTTCTAAACCGGTTGTTGGTGTTGGTCGTTTCACC
TCTCCGGACGTTATGGCGCGTATGGTTCGTCAGGGTGTTCTGGACTTCATCGGTTGCGCG
CGTCCGTCTATCGCGGACCCGTTCCTGCCGAAAAAAATCGAAGAAGGTCGTATCGAAGAC
ATCCGTGAATGCATCGGTTGCAACATCTGCATCACCGGTGACATGACCATGTCTATCTCT
CGTTGCACCCAGAACCCGACCTTCATGGAAGAATGGCGTAAAGGTTGGCACCCGGAACGT
ATGAACGCGAAAGGTGACTCTAACACCGTTCTGGTTGTTGGTGCGGGTCCGGCGGGTCTG
GAAGCGACCCGTGCGCTGTCTCTGCGTGGTTACGACGTTACCCTGGCGGAAGCGACCACC
ACCCTGGGTGGTCGTGTTGCGCGTGAACGTCTGCTGCCGGGTCTGTCTGCGTGGGGTCGT
GTTGTTGACTACCGTCAGTACCAGATCTCTCAGCGTACCAACGTTGAAACCTACTTCGAC
TCTCGTCTGACCGCGGAAGACGTTCTGGGTTTCGGTTTCGAACACGTTGCGATCGCGACC
GGTTCTCACTGGCGTCGTGACGGTGTTGCGCGTCAGCACGTTGTTCCGATGCCGATCGAC
CCGTCTATGACCGTTTGGACCCCGGACGACATCATGGCGAAAGTTCACCCGGAAAACCTG
TCTGGTAAAACCGTTGTTGTTTACGACGACGACCACTACTACATGGGTGGTGTTATGGCG
GAAGTTATGGCGAAAGCGGGTGCGAAAGTTATCCTGGTTACCTCTTCTGCGTACGTTTCT
GACTGGACCCGTAACACCCTGGAACAGGGTGCGATCCACGTTCGTCTGGACGACCTGGGT
GTTGACATCCGTCTGAACCGTGGTGTTACCGCGATCCGTGCGGGTGAAGTTGAAACCAAC
TGCGTTTACACCGGTAAACGTTCTGCGATCGGTTGCGACGCGGTTCTGATGGTTGCGTCT
CGTACCTCTGAAGACCAGCTGTTCAACGACCTGATCGCGCGTCAGGGTGACTGGCCGGAC
GCGGGTATCAAATCTGTTAAAATCATCGGTGACGCGGCGGCGCCGGCGCCGATCGCGTGG
GCGACCTACGCGGGTCACCGTTACGCGCGTGAACTGGACACCCCGGACATCGGTGACGAC
CTGCCGTTCCGTCGTGAAGTTACCCAGCTGGAACCGGCGTAA

Figure S7: DNA sequence used in this work for *E. coli* recombinant production of *Rhizobium Sp.* HDH (2142 bp), the codon usage, was optimized for *E. coli* transcription/translation. The sequence in bold is coding for the N-terminal 6x Histidine tag genetically added to the enzyme.

MGSSHHHHHHSSGLVPRGSHMRDNKYDILFEPVRIGPHIAKNRFYQVPHCNGGGYRDPSA

AAAMRGIKSEGGWGVIFTEQTEMHHTSEITPFIELRLWEDKDIPGLRRMSDAMKVHGALA

GIQLAYSGINGPNFYTKEVPLAPSALPIRTFTNDPVQARALDKQDIKNLRRWFVNAAKRS

KIAGFDLICLYGAHGFGIFQHFLSRATNQRTDEYGGSLENRSRFAREVVEDIKEAVGDTT

AITMRVSLDETIGELGFSNAEVREFVEMNANLPDLWDLAQGTWEDCSGPSRFKEEGAQEI

LVKGIRELSSKPVVGVGRFTSPDVMARMVRQGVLDFIGCARPSIADPFLPKKIEEGRIED

IRECIGCNICITGDMTMSISRCTQNPTFMEEWRKGWHPERMNAKGDSNTVLVVGAGPAGL

EATRALSLRGYDVTLAEATTTLGGRVARERLLPGLSAWGRVVDYRQYQISQRTNVETYFD

SRLTAEDVLGFGFEHVAIATGSHWRRDGVARQHVVPMPIDPSMTVWTPDDIMAKVHPENL

SGKTVVVYDDDHYYMGGVMAEVMAKAGAKVILVTSSAYVSDWTRNTLEQGAIHVRLDDLG

VDIRLNRGVTAIRAGEVETNCVYTGKRSAIGCDAVLMVASRTSEDQLFNDLIARQGDWPD

AGIKSVKIIGDAAAPAPIAWATYAGHRYARELDTPDIGDDLPFRREVTQLEPA

Figure S8: Amino acid sequence used in this work for *Rhizobium Sp.* HDHs (713 aa), the codon usage, was optimized for E. coli transcription/translation. The sequence in bold is coding for the N-terminal 6x Histidine tag genetically added to the enzyme.

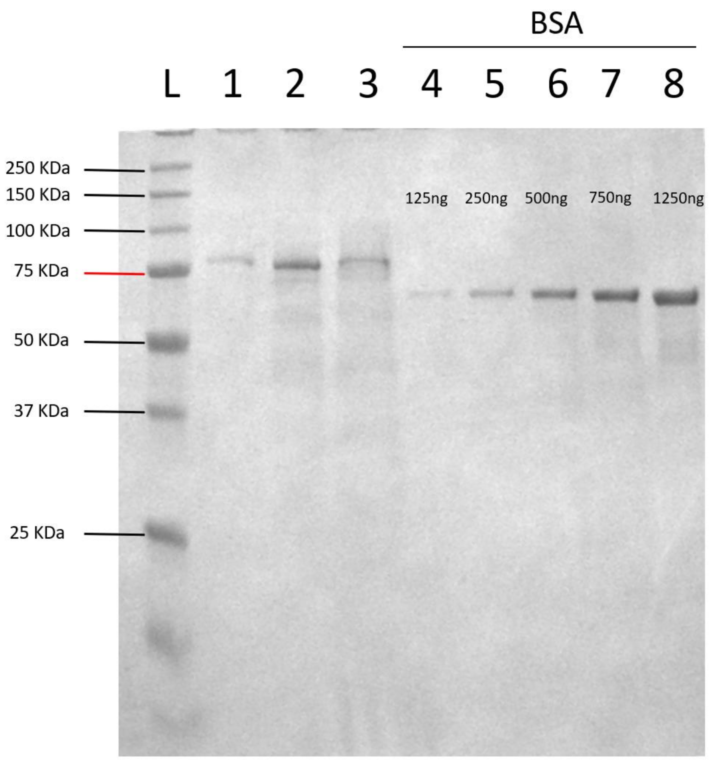

Figure S9: SDS-PAGE analysis of purified HDH WT Rsp (1), Phe72Thr (2), and Asn110Val (3) and known Bovine Serum Albumin (BSA) of known concentration ((4) to (8): 0.05, 0.1, 0.2, 0.3, 0.5 mg/mL representing 125, 250, 500, 750 and 1250 ng protein per well, respectively). The relationship between pixel density in the image and the amount of protein in each band was obtained using the software Image J. The protein quantification through interpolation for each purified HDH variant sample was calculated to be 0.091 mg/mL for WT Rsp HDH, 0.226 mg/mL for variant Phe72Thr and 0.178 mg/mL. These values were adjusted by volume in assay and dilution factor for specific activity calculations of the corresponding variant.

Supporting References

1. Tsutsumi, M., et al., *Thermodynamic redox properties governing the half-reduction characteristics of histamine dehydrogenase from Nocardioides simplex.* Bioscience, biotechnology, and biochemistry, 2008. **72**(3): p. 786-796.
2. Huang, C.-H., et al., *Crystal structure of glucooligosaccharide oxidase from Acremonium strictum: a novel flavinylation of 6-S-cysteinyl, 8α-N1-histidyl FAD.* Journal of Biological Chemistry, 2005. **280**(46): p. 38831-38838.
3. Stourac, J., et al., *Caver Web 1.0: identification of tunnels and channels in proteins and analysis of ligand transport.* Nucleic acids research, 2019. **47**(W1): p. W414-W422.
4. Ashkenazy, H., et al., *ConSurf 2016: an improved methodology to estimate and visualize evolutionary conservation in macromolecules.* Nucleic acids research, 2016. **44**(W1): p. W344-W350.
5. Lua, R.C., et al., *UET: a database of evolutionarily-predicted functional determinants of protein sequences that cluster as functional sites in protein structures.* Nucleic acids research, 2016. **44**(D1): p. D308-D312.
